# Mandatory Preemptive Skin Testing and Its Impact on Penicillin Allergy Labeling Across Different Healthcare Settings in Mainland China and Hong Kong

**DOI:** 10.1111/all.70065

**Published:** 2025-09-24

**Authors:** Weihong Shi, Lanting Wang, Shengan Chen, Philip Hei Li, Xiaoqun Luo

**Affiliations:** ^1^ Division of Rheumatology and Clinical Immunology, Department of Medicine, Queen Mary Hospital The University of Hong Kong Hong Kong SAR China; ^2^ Division of Rheumatology and Clinical Immunology, Department of Medicine University of Hong Kong‐Shenzhen Hospital Shenzhen China; ^3^ Department of Allergy and Immunology Huashan Hospital Affiliated to Fudan University Shanghai China; ^4^ Department of Dermatology Huashan Hospital Affiliated to Fudan University Shanghai China; ^5^ Research Center of Allergy and Disease Fudan University Shanghai China

To the Editor,

Mislabeled penicillin allergies restrict appropriate antibiotic use, contributing to antimicrobial resistance and reduced quality of life [1, 2]. In Mainland China, mislabeling has been largely driven by a national policy mandating preemptive penicillin skin testing (PST) with intradermal benzylpenicillin prior to all prescriptions [3]. In contrast, PST in Hong Kong is selectively performed among patients with a history of previous reactions [4]. This difference provides a unique opportunity to examine how allergy labels arise under differing healthcare policies and to inform future delabeling strategies [5]. Therefore, we conducted this regional study in Mainland China and Hong Kong to evaluate how differing policies affect penicillin allergy mislabeling.

We recruited patients labeled with penicillin allergy from 22 tertiary hospitals in Shanghai (Mainland China) and from the Drug Allergy Delabeling Initiative in Hong Kong between December 2022 and December 2024, using a retrospective review of medical records and registry data (Data S1) [4]. Categorical variables were compared using the chi‐squared test or Fisher's exact test. *p* values < 0.05 were considered significant. Statistical analyses were performed using R v4.5.0. All patients provided informed consent, and the study was approved by the Institutional Review Boards of the University of Hong Kong/Hospital Authority Hong Kong West Cluster and Huashan Hospital of Fudan University.

A total of 2069 (Mainland China: 1277, Hong Kong: 792) patients were recruited. The female:male ratio was 2.39:1 with no regional difference (*p* = 0.10). Patients in Mainland China were younger, with a higher proportion aged ≤ 50 years (67.03% vs. 27.78%, *p* < 0.001). This age difference may reflect the long‐term impact of mandatory PST: mislabeling in younger patients accumulates with age, leading to restricted prescribing and reduced healthcare utilization in later life [6]. In Mainland China, 77.37% (988/1277) of labels resulted from preemptive PST, compared to only 2.27% (18/792) in Hong Kong, all of whom were originally labeled in the Mainland (Figure 1). After excluding patients labeled solely by preemptive PST and without documented reactions, significant differences in manifestations persisted between the regions (Table 1). Mucocutaneous manifestations accounted for the majority (89.49%) in Hong Kong, compared to only 38.81% in Mainland China (*p* < 0.001). In contrast, there were higher rates of cardiovascular manifestations in Mainland China (47.76% vs. 6.20%, *p* < 0.001). Delabeling was not performed in Mainland China, while the overall delabeling rate in Hong Kong was 91.43% (704/770). There were no differences in delabeling rates regardless of the reason for initial labeling: 91.17% (640/702), 94.12% (16/17), and 94.12% (16/17) for those with prior history, preemptive PST, and other reasons, respectively.

**FIGURE 1:**
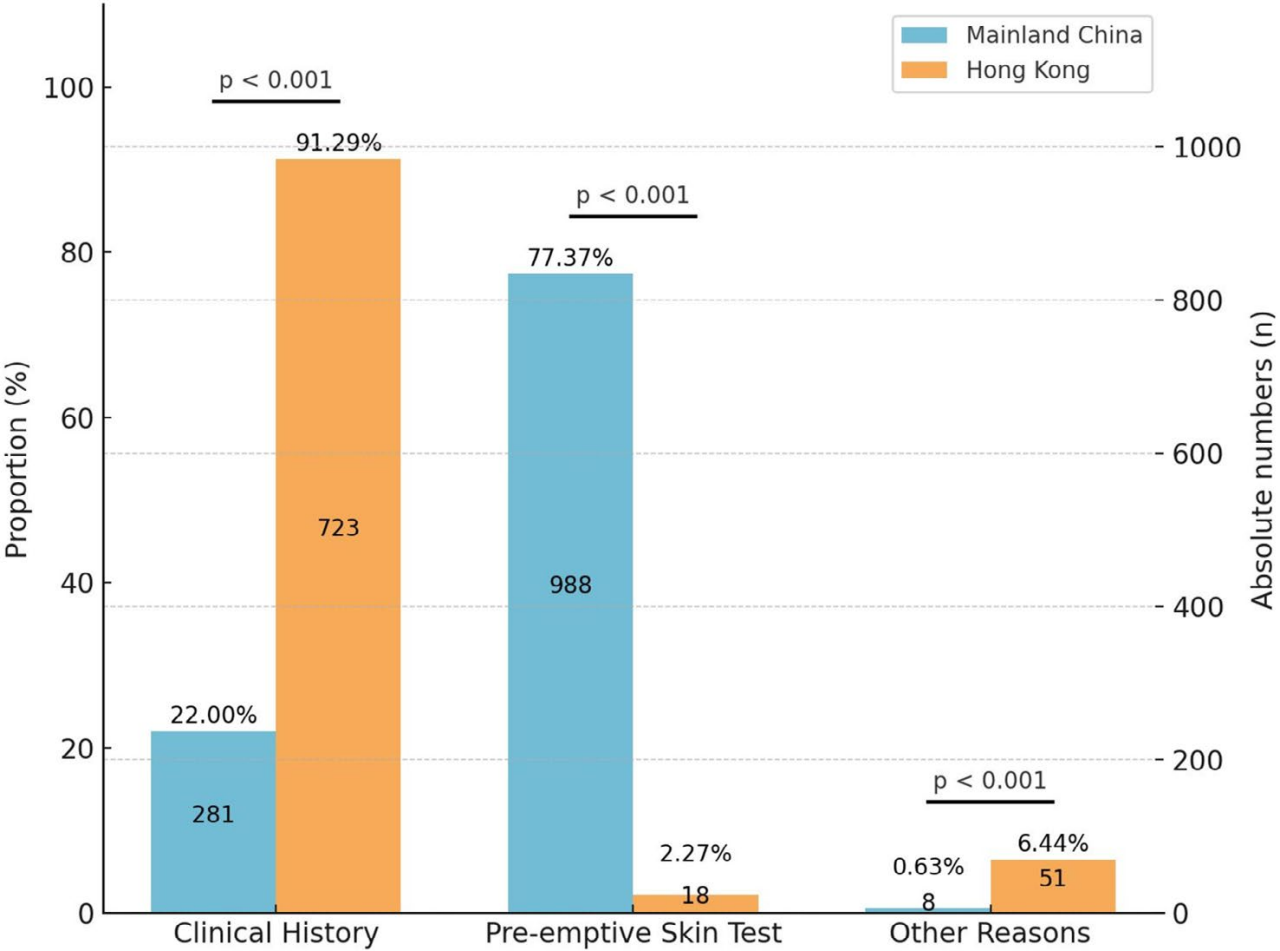
Distribution of reasons for penicillin allergy labeling in Mainland China and Hong Kong. Bar heights indicate proportion of total cases; values inside bars indicate absolute numbers (n). “Other Reasons” include family history of penicillin allergy, unverified patient or caregiver self‐reporting, historical labels without identifiable history or clinical basis, and concerns about cephalosporin cross‐reactivity. *p* Values represent statistical significance between regions for each labeling source.

**TABLE 1:** Reported clinical manifestations of index reaction among those patients reporting a history of previous reaction to penicillins.

Manifestation	Mainland China (*N* = 67)	Hong Kong (*N* = 742)	OR (95% CI)	*p*
Mucocutaneous	26 (38.81%)	664 (89.49%)	0.07 (0.04–0.13)	< 0.001
Respiratory	4 (5.97%)	24 (3.23%)	1.90 (0.64–5.65)	0.280
Cardiovascular	32 (47.76%)	46 (6.20%)	13.83 (7.87–24.33)	< 0.001
Others^a^	5 (7.46%)	8 (1.08%)	7.40 (2.35–23.30)	0.003

^a^ “Others” include nonspecific systemic symptoms, such as fever, chills, headache, and joint pain.

This regional observational study highlights the profound influence of differing healthcare policies on allergy mislabeling. In contrast to Hong Kong, over 77% of penicillin allergy labels in Mainland China originated from preemptive PST. Despite the nearly identical ethnic background of the cohorts, the mandatory PST policy in Mainland China likely drives the high rate of penicillin allergy mislabeling. Interestingly, even after excluding patients labeled by preemptive PST, Mainland China still showed a higher proportion of systemic (nonmucocutaneous) reactions. This marked difference may be influenced by ascertainment bias yet still highlights the failure of preemptive PST to prevent systemic reactions and the potential for false‐negative results. Instead, widespread mislabeling further drives unnecessary penicillin avoidance, contributing to higher antimicrobial resistance and lower inpatient penicillin use in Mainland China [3].

This study had limitations, including limited clinical data and specific penicillin agents in the Mainland China cohort, precluding further comparisons. The Mainland China cohort was also limited to Shanghai.

In conclusion, our study accumulates further evidence confirming the harm associated with routine preemptive PST, highlighting the urgent need for evidence‐based practices and global collaboration to address antibiotic allergy mislabeling and optimize antimicrobial use.

## Author Contributions

Philip Hei Li conceived the study and performed a critical revision of the manuscript. Weihong Shi, Lanting Wang, and Shengan Chen contributed to data collection, analysis, and drafting of the manuscript; Weihong Shi additionally performed visualization and manuscript review and editing. Xiaoqun Luo contributed to conceptualization, supervision, and data interpretation.

## Conflicts of Interest

The authors declare no conflicts of interest.

## Supporting information


**Data S1:** all70065‐sup‐0001‐Supinfo1.docx.

## Data Availability

The data that support the findings of this study are available on request from the corresponding author. The data are not publicly available due to privacy or ethical restrictions.
