# Supplementary material for: Mandatory Preemptive Skin Testing and Its Impact on Penicillin Allergy Labeling Across Different Healthcare Settings in Mainland China and Hong Kong
Source: Allergy. 2025 Sep 24;81(5):1851–3. doi: 10.1111/all.70065 (PMC13139797; doi:10.1111/all.70065)
Supplement: Supplementary file 1 — Data S1: all70065‐sup‐0001‐Supinfo1.docx. [file ALL-81-1851-s001.docx]

**List of Participating Institutions**

**Mainland China (Shanghai Cohort)**

1. Department of Allergy & Immunology, Department of Dermatology, and Department of Otorhinolaryngology-Head and Neck Surgery, Huashan Hospital Affiliated to Fudan University
2. Department of Pulmonary and Critical Care Medicine, Department of Dermatology, Ruijin Hospital Affiliated to Shanghai Jiao Tong University School of Medicine
3. Department of Skin & Cosmetic Research, Shanghai Skin Disease Hospital
4. Department of Dermatology, Pudong New Area People's Hospital
5. Department of Geriatrics, Department of Otolaryngology-Head and Neck Surgery, Xinhua Hospital Affiliated to Shanghai Jiao Tong University School of Medicine
6. Department of Respiratory Medicine, Children's Hospital of Fudan University
7. Department of Respiratory Medicine, Department of Dermatology, Shanghai Children's Hospital
8. Department of Otorhinolaryngology Head and Neck Surgery, Department of Pulmonary and Critical Care Medicine, Tongji Hospital Affiliated to Tongji University
9. Department of Pulmonary and Critical Care Medicine, Zhongshan Hospital Affiliated to Fudan University
10. Department of Paediatrics, Shanghai General Hospital
11. ENT institute and Department of Otorhinolaryngology, Eye & ENT Hospital Affiliated to Fudan University
12. Department of Otolaryngology-Head and Neck Surgery, Shanghai Sixth People's Hospital Affiliated to Shanghai Jiao Tong University School of Medicine
13. Pediatric Department, Shanghai Jiading District Nanxiang Hospital
14. Department of Dermatology, Shanghai Putuo District People's Hospital
15. Department of Dermatology, The Ninth People's Hospital Affiliated to Shanghai Jiao Tong University School of Medicine
16. Department of Dermatology, Shanghai Tenth People’s Hospital
17. Department of Dermatology, Renji Hospital Affiliated to Shanghai Jiao Tong University School of Medicine
18. Department of Dermatology, Shanghai Fourth People's Hospital
19. Department of Dermatology, Shanghai Fengxian District Central Hospital
20. Department of Otorhinolaryngology, Shanghai Xuhui District Central Hospital
21. Department of Dermatology, Longhua Hospital Affiliated to Shanghai University of Traditional Chinese Medicine
22. Department of Dermatology, Shanghai TCM Integrated Hospital Affiliated to Shanghai University of Traditional Chinese Medicine

**Hong Kong Special Administrative Region, China (Hong Kong Cohort & Registry)**

1. Division of Rheumatology & Clinical Immunology, Department of Medicine, Queen Mary Hospital, University of Hong Kong, Hong Kong, China
2. Division of Infectious Diseases, Department of Medicine and Geriatrics, Princess Margaret Hospital, Hong Kong, China
3. Division of Infectious Diseases, Department of Medicine, Queen Elizabeth Hospital, Hong Kong, China
4. Division of Infectious Diseases, Department of Medicine & Therapeutics, Prince of Wales Hospital, Hong Kong, China
